# Proposal of Platelet Anti-aggregation in Transplant Renal Artery Stenosis

**DOI:** 10.7759/cureus.89265

**Published:** 2025-08-02

**Authors:** Jorge Arellano Soto, Enrique Amador Ortega Santamaria, Felix Adrian Vergara Martínez, Ramón Espinoza Pérez, Francisco J Avelar Garnica, María Alejandra Morinelli Astorquizaga, Adriana Irais Flores Juárez, Samuel Hernández Ballesteros, Juan Figueroa García, Juan Carlos H Hernández Rivera

**Affiliations:** 1 Diagnostic and Therapeutic Imaging Department, Hospital de Especialidades Centro Medico Nacional (CMN) Siglo XXI, Mexico City, MEX; 2 Kidney Transplant Unit, Hospital de Especialidades Centro Medico Nacional (CMN) Siglo XXI, Mexico City, MEX; 3 Training and Continuing Education Department, Hospital de Especialidades Centro Medico Nacional (CMN) Siglo XXI, Mexico City, MEX; 4 Medical Research Unit in Nephrological Diseases, Hospital de Especialidades Centro Medico Nacional (CMN) Siglo XXI, Mexico City, MEX

**Keywords:** angioplasty, anti-aggregation therapy, hypertension, interventional, proposal, transplant renal artery stenosis

## Abstract

Introduction

Kidney transplant (KT) offers the best renal replacement therapy (RRT), not without being exempt from medical-surgical complications, highlighting those that impact graft function with elevated serum creatinine compounds, such as transplant renal artery stenosis (TRAS), and this study, in turn, has presented some gaps in knowledge, such as anti-aggregation therapy, essentially after the interventionist handling activity. The objective of this document is to propose a platelet anti-aggregation scheme and show the behavior of a series of cases, with a review of the literature.

Materials and methods

This is a case report of patients with TRAS for five years and six months; patients with a clinical imaging diagnosis of TRAS were included, who were managed with intervention and follow-up.

Results

In the study, a prevalence of 1.35% (11 of 813) of confirmed cases treated with angioplasty was documented; of the 11 cases, one had restenosis, and another had a thrombotic event requiring reintervention. Antiplatelet therapy was used in 11 patients: one patient was diagnosed at examination, three on the day of angioplasty, and seven cases within seven days after angioplasty. The duration of maintenance with dual or single antiplatelet therapy has not been standardized; in our cases, dual therapy lasted 1-9 months, followed by single antiplatelet therapy.

Conclusions

The interventional treatment of TRAS is safe; however, there is variability, and antiplatelet therapy is not standardized. This antiplatelet therapy should be determined based on risk factors, stenosis characteristics, and comorbidities. Based on all of this, this study is proposed to standardize antiplatelet therapy.

## Introduction

Kidney transplant (KT) is considered to be the best option of kidney replacement therapy (KRT) [1], although it is not without complications, which are classified as medical and surgical; within the surgical ones, they are divided as intraoperative and postoperative by temporality, the nature of complications, urology, or blood vessel injuries such as dissection, thrombosis, pseudoaneurysm, and arterial stenosis [2,3]. Among the vascular complications, transplant renal artery stenosis (TRAS) is described as the most common one, with incidences of 1%-22% of all patients, occurring from three months to two years after transplantation and in some cases up to three years, with the first six months being more common [2-5]. The importance of knowing this entity lies in that it affects the long-term survival of the patient and of the graft, in addition to being the potential curable cause of refractory hypertension and graft dysfunction. It may be asymptomatic or manifest as refractory hypertension to treatment, peripheral edema, pulmonary acute edema “flash type,” and the elevation of serum creatinine [3-5].

TRAS can be defined as the decrease of the artery caliber of greater than 50% demonstrated by angiography, with atherosclerosis being the predominant cause in these patients [3,6]; the treatment can be divided into three: (1) medical management (antihypertensives, lipid-lowering agents, and in some cases platelet anti-aggregation) [6-9], (2) interventional management (angioplasty with or without stent placement plus medical treatment), and (3) revascularization [6,9]. Some studies compare the medical interventional management; among them are the ShorT stay Aneurysm Repair (STAR) study [10], the ASTRAL trial [11], the CORAL study [12], and finally a metanalysis [13]; if the interventional management has no benefits, the following are evaluated as primary outcomes: death due to cardiovascular or renal cause, acute myocardial infarction, stroke, and progressive kidney disease; the need of KRT; improvement in renal function; and the incidence of malignant or refractory hypertension, acute pulmonary edema, and cardiac failure [10-13]. Some of these studies have been object of reviews and discussions, in which their methodologies, the inclusion criteria, the exclusion of some patients with clinical presentations of critical stenosis, and patients with stenosis of <50% are questioned [9-13]; one of the most important aspects is that population with stenosis of >70% was smaller [8,9]; in the evaluation of the outcomes, no difference was shown, although contradictory results were found when evaluating which type of treatment was best [9-12].

The new guidelines and reviews for the renovascular hypertension management indicate the performing transluminal angioplasty with or without stent in patients with stenosis of >70% is associated with resistant hypertension or recent-onset acute flash pulmonary edema, acute heart failure, the rapid deterioration of renal function, intolerance to angiotensin-converting enzyme inhibitors (ACEi) or angiotensin receptor blockers (ARBs), and KRT; other indications include acute renal injury in acute or high-grade stenosis, patients with KT, extrinsic vascular compression, and monorenal stenosis [7-9,14-16]. Native kidney stenosis with interventional management is complemented by the physician; this is due to the decrease in the risk of cardiovascular death, cardiac remodeling, and cerebral vascular events [8,17-20]. The duration of dual antiplatelet therapy (DAPT) and simple therapy has not yet been established. Anti-aggregation therapy can be considered upon the suspicion of stenosis [12] or one day prior to angioplasty [10,17-19]. Regarding the duration of DAPT, managements of one month [17-19], 1-3 months [8], six weeks [21], or up to six months [22] were found; once this period has ended, most recommended continuing with the simple anti-aggregation therapy for at least 12 months or indefinitely, usually with acetylsalicylic acid (ASA) [21-24]. Part of the management used in TRAS derives from the knowledge obtained in native kidney stenosis and coronary and carotid catheterizations, among others [8,9,14-16]. Due to these problems, this study seeks to suggest an anti-aggregation therapy in four moments: (a) primary prevention post-KT, (b) diagnosis, (c) peri-angioplasty, and (d) maintenance, for decision-making to initiate simple anti-aggregation therapy, the duration of the DAPT, and the maintenance of simple antiplatelet therapy.

## Materials and methods

Design

This is a descriptive, analytical, longitudinal study of a retrospective cohort of patients with KT.

Patients

The patients were identified as part of a retrospective study from a Mexican cohort of kidney transplant receptors from October 2018 to March 2024, which include 813 KT patients; the inclusion criteria were KT patients with age of ≥18, with confirmed stenosis of at least 50% in angiotomography, and managed with angioplasty and/or stent; the TRAS diagnosis was under clinical suspicion of hypertension of recent diagnosis or difficult to control and/or elevated serum creatinine and critical presentations; the conventional algorithm was initially with Doppler ultrasound of the graft having a suspicion of stenosis with peak systolic velocity (PSV) of >200 cm/second, followed by confirmatory angiotomography and compared with angiography. The exclusion criteria were patients who could not corroborate the grade of stenosis in at least one image studio and whose medical records were not located physically or virtually.

Data collection

When identifying cases, relevant pathological personal history, KT date, TRAS data, angiography results, angioplasties, and stent characteristics are recorded. Anti-aggregation therapy is reviewed prior to diagnosis, at the time of intervention, during hospital stay, and on an outpatient basis, with at least one year of follow-up. The potential complications derived from the procedure are also documented. The National Committee for Scientific Research of the Mexican Institute of Social Security (IMSS) issued approval R-2022-785-019.

Statistics

Descriptive statistics were performed for quantitative variables. The Shapiro-Wilk normality test was applied, with mean or median as appropriate to its distribution; for dispersion measures, standard deviation or ranges were applied as appropriate. In the case of the percentage of stenosis, it also includes the minimum and maximum. For qualitative variables, frequencies and percentages were represented. The baseline characteristics and a chart of the cases are made with the most important events; the statistical package SPSS version 27 (IBM Corp., Armonk, NY) is used.

## Results

Of the 813 patients, 11 could be identified who met the inclusion criteria; the baseline characteristics, data related to the diagnosis, and interventional treatment are presented in Table 1.

**Table 1:** Patient characteristics *Both patients had creatinine elevation, and one patient presented with acute pulmonary edema ^Ŧ^Median, minimum, and maximum ^‡^Restenosis at six months, managed with one stent CKD, chronic kidney disease; KRT, kidney replacement therapy

Baseline characteristics	n=11
Men	8 (72.7%)
Women	3 (27.2%)
Smoking history	1 (9%)
History of hypertension	11 (100%)
Age	47.2 (32-66)
Cause of CKD	
Diabetic nephropathy	2 (18.1%)
Polycystic kidney disease	2 (18.1%)
Unknown etiology	5 (54.5%)
Lupic nephropathy	1 (9%)
Postinfectious glomerulonephritis	1 (9%)
KRT	
Hemodialysis	8 (72.7%)
Peritoneal dialysis	3 (27.2%)
Primary cause of suspicion	
Refractory or resistant hypertension	6 (54.5%)
Fluid overload*	2 (18.1%)
Creatinine elevation	2 (18.1)
Ultrasound findings	1 (9%)
Number of antihypertensive drugs at diagnosis	
5	3 (27.2%)
4	2 (18.1)
3	3 (27.2%)
2	2 (18.1)
1	1 (9%)
Stenosis approach	
Percent of stenosis in angiotomography^Ŧ^	70 (50-92)
Percent of stenosis in angiography^Ŧ^	73 (45-90)
1 stent	9 (82%)
2 stents	1 (9%)
Only angioplasty^‡^	1 (9%)

Anti-aggregation

All 11 cases underwent angioplasty; after management in the procedure room, it was confirmed that blood flow was immediately restored between 85% and 100%. Regarding anti-aggregation therapy at the diagnosis of TRAS or prior to angioplasty, only 100 mg of ASA was used in one case 11 months prior (9%), and clopidogrel was added at discharge on day 4. Three patients (27.2%) began anti-aggregation therapy the day of the procedure, one of them presenting restenosis at six months; in the second intervention, in addition to the placement of the stent, DAPT was started on day 7 until the present day. One patient on day 1 post procedure (9%), one patient on day 3 (9%), and three patients on day 4 (27.2%). One patient was discharged on day 4 without anti-aggregation therapy; two days later, he presented with intense pain, documenting arterial thrombosis of the graft. Performing a new angioplasty, in addition receiving 3000 IU of heparin, he presented flow to graft; from days 7 to 10, receives enoxaparin and tirofiban indicated by hematology; and begins the DAPT on day 11; for complications, he presented two arteriovenous fistulas and exacerbation of the function of the graft that required hemodialysis, which he did not recover function, and initiating the protocol for a second transplant. DAPT was maintained for three months; later, both were suspended.

One patient did not receive anti-aggregation therapy (9%); in this case, creatinine decreased from 5.8 to 1.9 mg/dL after angioplasty; however, graft function associated with two multiresistant urinary tract infection (UTI) events was complicated, the first in the following two weeks and the second with a UTI and pneumonia associated with mechanical ventilation, which led to acute graft disfunction, requiring KRT with hemodialysis without recovery and the loss of graft four months after angioplasty. DAPT maintenance was performed in five patients (45.4%) with a mean of 7.16 months (three for six months, one for seven months, and two for nine months); another case is scheduled for six months with DAPT. Other patients only use one antiaggregant or do not have any; of these patients, only one used clopidogrel management, one patient started ASA and has maintained its use for 12 months, and one case without anti-aggregation therapy presented restenosis at six months; in a second angioplasty, a stent was placed, and DAPT was maintained for six months. Consistently in all cases, after the discontinuation of clopidogrel, ASA is maintained, where one of the patients, who was even the first case in our center, is currently on ASA treatment for four years. The restenosis percentage in our cases was 9%, consistent with the 5%-22% found in the literature. This may vary according to the type of procedure performed and anatomical location and is higher in patients without a stent. Data related to the individual medical history, interventional management of stenosis, DAPT initiation, duration, single antiaggregant treatment, and complications are presented in Table 2.

**Table 2:** Intervention SAH, systemic arterial hypertension; CKD, chronic kidney disease; KT, kidney transplant; SLE, systemic lupus erythematosus; ASA, acetylsalicylic acid; DAPT, dual antiplatelet therapy; CTA, computed tomography angiography; SC, subcutaneous; PSV, peak systolic velocity

Case	Medical history	Clinical manifestations	Time between KT and diagnosis	Intervention	Percentage of stenosis	Anti-aggregation postangioplasty	Complications
1	Male, 39 years old, SAH (2015), non-determined CKD (2017), and KT (2023-06-19)	SAH-resistant	Two months and 16 days	2023-07-27: balloon angioplasty	CTA, 53% in the anastomosis; angiography, 66% in the anastomosis, angioplasty without stent	Without anti-aggregation therapy. Enoxaparin 40 mg. SC of 12 hours. Post procedure for two days	Right femoral artery aneurysm and restenosis
2	Male, 51 years old, SAH (2007), left nephrectomy (2022), polycystic kidney disease (2010), and KT (2023-05-01)	SAH-resistant	Two months and four days	2023-07-19: Herculink stent	CTA, 70% in the anastomosis; angiography, 90% in the anastomosis	DAPT: clopidogrel 75 mg and ASA 100 mg/24 hours for six months. Maintenance: ASA 100 mg every 24h	-
3	Male, 41 years old, left nephroureterectomy (2022), CKD from renal hypoplasia (2019), and KT (2022-09-29)	SAH-resistant	Two months and 23 days	2023-01-25: RX Herculink Elite stent, Abbott, 5×18 mm	CTA, 60% in the anastomosis; angiography, 55% of stenosis in the anastomosis	DAPT: clopidogrel 75 mg and ASA 150 mg every 24 hours for six months. Maintenance: continues with ASA 150 mg every 24 hours	-
4	Male, 54 years old, SAH (2001), left radical renal nephrectomy for kidney cancer (2022), CKD non-determined (2017), and KT (2022-04-11)	SAH-resistant	Three months	2022-08-17: Bently stent, 5×28 mm	CTA, 92% in the anastomosis; angiography, stenosis at the level of the anastomosis, renal artery of 3.44 mm	DAPT: clopidogrel 75 mg and ASA 150 mg each 24 hours. Starting the day 0 for seven months. Maintenance: ASA 100-150 mg every 24 hours	-
5	Male, 35 years old, SAH (2019), CKD non-determined (2019), and KT (2022-01-11)	Creatinine increase	Six months and eight days	2023-03-13: two Herculink stents, 5 Fr 0.071/1.80 mm	CTA, 70% in the anastomosis, length of 7.8 mm; angiography, bending at the level of the anastomosis and at 3 cm. Two stents are placed	ASA 150 mg every 24 hours	Hematoma in the right inguinal region
6	Female, 38 years old, SAH (2012), CKD for renal hypoplasia (2011), and KT (2016-07-07)	SAH-resistant	Three years and 10 months	2020-08-24: balloon-mounted stent, 5×23 mm	CTA, 91% with a length of 17.1 mm; angiography, stenosis in the first third of the renal artery	In-hospital management: enoxaparin and tirofiban on day 7. DAPT: clopidogrel 75 mg and ASA 150 mg on day 11 continue for three months. Anti-aggregation suspended	Thrombosis of the renal graft artery treated with thrombectomy and fibrinolysis. Graft loss due to thrombosis. Documentation of two arteriovenous fistulas
7	Female, 19 years old, SAH (2012), CKD non-determined (2012), and KT (2015-04-01)	Increase in antihypertensive drugs	Three years and four months	2018-10-02: variable diameter stent, 7×15 mm	CTA, 84% at the middle third of the renal artery; angiography, >80% at the middle third	DAPT: ASA 150 mg and clopidogrel 75 mg every 24 hours. Begins on day 1 and continues for nine months. Maintenance: ASA 150 every 24 hours	-
8	Male, 63 years old, type 2 diabetes (1998), SAH (2014), granulomatous hepatitis and diabetic nephropathy (2014), and KT (2021-06-20)	Detection of the increase of PSV in consulting ultrasound	Two years and two months	2023-08-04: BeSmooth stent, 6 Fr 6×18 mm	CTA, 70% in the anastomosis, in post-stenotic region multiple calcified atheroma; angiography, 80% at the level of the anastomosis	Previous antiplatelet therapy: started at diagnosis with ASA 11 months prior to angioplasty. DAPT: clopidogrel 75 mg and ASA 150 mg every 24 hours. DAPT for six months. Maintenance: continue with ASA 100 mg every 24 hours	-
9	Female, 42 years old, SAH (2021), SLE (2020), left ovarian tumor (2019), lupic nephritis (2021), and KT (2023-02-10)	Fluid overload, dyspnea, and an increase in serum creatinine	One month and 12 days	2023-04-04: BeSmooth stent, 6×18 mm	CTA, 50% in the anastomosis; angiography, 45% in the anastomosis	Without anti-aggregation therapy in-hospital or outpatient	Hematoma in the right inguinal region
10	Female, 49 years old, polycystic kidney disease (2002), SAH (2002), nephrectomy (2015), and KT (2023-09-14)	Increase in serum creatinine	Four months and 10 days	2024-01-29: BeSmooth stent, 5×23 mm	Angiography, 70% in the anastomosis and 20 mm in length. Two angioplasties were performed; a stent was placed, with a resolution of 80% of the stenosis	Clopidogrel 75 mg each 24 hours on day 3 after angioplasty. Without DAPT	Vasospasm in segmental branches in hemodynamics treated with manual aspiration and nimodipine
11	Male, 67 years old, SAH (2008), hyperprolactinemia and diabetic nephropathy (2018), and KT (2022-06-10)	Acute pulmonary edema, fluid overload, and creatinine increased	Eight months and 24 days	2024-03-06: Bentley stent, 6×20 mm	CTA, 74% in the anastomosis; angiography, 64% in the anastomosis	Clopidogrel 75 mg and ASA 100 mg every 24 hours. Starting on day 0. Continues with DAPT	-

## Discussion

This is a study on cases of TRAS, with a prevalence of 1.35% (11 of 813 transplant recipients); due to the fact that there is no algorithm that handles anti-aggregation therapy, there is no consistency in its approach, despite the fact that there are great series related to stenosis in native kidneys, such as the CORAL studies [12] with 947 patients divided in two groups, a group with medical therapy and a group complemented with interventionism (1:1), where anti-aggregation with atorvastatin is used and the time and dosage are not specified, and the ASTRAL study [11] with 806 patients separated in two groups (1:1), where both received antihypertensives, lipid-lowering agents (statins), and anti-aggregation (ASA) without specifying neither dosage nor duration; this makes us see the little importance that has been given to postangioplasty anti-aggregation therapy, although the primary objective of both studies was the clinical and biochemical behavior; the few literatures found addressed postangioplasty management and mentioned the use of ASA 100 mg indefinitely and clopidogrel 75 mg during the first month [19]. However, among the main drawbacks of angioplasty, there are major complications related to bleeding, such as hematomas or pseudoaneurysms, but without surgical management, where there should be the possibility of categorization of patients with or without the risk of bleeding.

During the review of anti-aggregation therapy in TRAS, a research was carried out in PubMed about the MeSH terms “transplant renal artery stenosis” with “antiaggregant,” “anticoagulation,” “aspirin,” or “DAPT,” alone or together. However, no related articles were found; given this, we consider it of vital importance to make a proposal of anti-aggregation therapy considering the patient’s own risk factors and of the graft, the bleeding risk, anatomy, injury characteristics, and the major or minor complications of the angioplasty that may occur to determine the duration of DAPT. Some articles and guidelines recommend, in manuscripts directed at native kidneys, medical management with antiplatelet and lipid-lowering agents from the diagnosis of renal stenosis, which may or may not be accompanied by angioplasty with or without a stent [6,8,9]; in those managed by angioplasty with or without a stent, DAPT is started, mainly with ASA 100 mg and clopidogrel 75 mg every 24 hours where there is variation in time: some recommend management for one month [17-19], others for 1-3 months [8], and another for up to six months and then continue only with ASA. This information is for patients with native kidney affection and those not focused on the management of patients with TRAS.

In primary cardiovascular prevention, the ASCEND study [24] included patients with diabetes without an established cardiovascular disease, a decrease in cardiovascular events, and an increase in complications such as bleeding; subsequently, the ARRIVE [25] and ASPREE [26] studies found an increased risk of bleeding without a decrease in major cardiovascular events. In the ESC 2021 guide, ASA is considered in patients with type 2 diabetes and high or very high risk as a class IIb recommendation [27]. In 2022, the USPSTF, with a grade of evidence C, only considers ASA in adults aged 40-59 years with a 10% risk and that ASA should be individualized [28]. In general, the few recommendations about primary prevention propose that its use should be individualized according to the cardiovascular risk; seeking that specific patients benefit, platelet phenotyping and the usage of a model for the study in primary prevention are insisted [27-30]. Antiplatelet therapy for antiretroviral therapy (ART) could be started from the moment of diagnosis [8,21]. An interesting approach is based on a prospective, randomized, controlled study that proposes the use of 100 mg ASA as primary prevention, two weeks after the ART, with a difference in the incidence of confirmed ART of 2.8% in the ASA group and 11.6% in the control group [23]. In addition, in a retrospective study of patients from 1975 to 2001 who started ASA two years after transplantation, they had higher graft survival, slower creatinine increase, and lower proteinuria and hematuria; these results were better in the subgroup with longer treatment duration [31].

In a meta-analysis [32], studies comparing ASA and placebo were evaluated, finding a decrease in the risk of graft failure, thrombosis, major cardiovascular events, and mortality, without a decrease in delayed function or rejection [32]. In a retrospective cohort of three groups, where they had a control group without ASA, a second group that started ASA two weeks after KT, and a third group at three months after KT, stenosis was confirmed in the control group at 13.1%, 13% in the early group, and 3.1% in the late group; this was associated with a slight increase in the risk of bleeding (13.7% versus 8.7%) [33-35]. This work proposes a comprehensive treatment beginning with ASA as primary prevention, associated with statins, considering initiation when the graft presents function, individualizing the characteristics of the patient as bleeding risk, events, and surgical incidents that may contraindicate early use, complications during hospitalization, and follow-up. ASA was continued for three months, and then, a graft ultrasound was performed to determine who present suggestive TRAS data and complement it with computed tomography (CT) angioplasty, where these data are not submitted and anti-aggregation therapy may be suspended and followed up with ultrasound according to the protocols of each hospital; in the case of the patients who subsequently present a TRAS diagnosis, it is recommended to initiate ASA with the suspicion or diagnosis of TRAS. This is a part of an analysis of primary prevention [23,27-35].

Regarding the duration of DAPT, we propose a 3-6-month scheme. We do not have a score that allows us to determine which patients are candidates for three or six months; it is necessary to consider who will benefit more with a conventional therapy. These factors, added to the cardiovascular and bleeding risk, can limit the duration of DAPT. Since there is no validated score for these patients, important points were collected that increase the risk of bleeding in multiple interventional procedures: the use of nonsteroidal anti-inflammatory drugs, steroid doses, serotonin reuptake inhibitors, people over 75 years, bleeding that required transfusion or hospitalization in the previous six months, cirrhosis, glomerular filtration rate of <59 mL/minute, hemoglobin of <11 mg/dL, platelets of <100000 μL, and invasive procedures or recent trauma [32,36-38]. The choice of the duration of DAPT should take into consideration these factors of the risk of bleeding, together with the risk of stenosis, restenosis, and thrombosis to identify which patients will benefit from each therapy.

Factors to be evaluated and taken into consideration for the risk of stenosis were divided into three groups. The first is those that may interfere with the genesis and progression of TRAS: the vascular characteristics, the type of anastomosis, atherosclerosis in the donor’s kidney, concomitant diseases such as diabetes, cold ischemia time, deceased donor, and delayed graft function [3,5,18,21]. Historically, cytomegalovirus infection has been related as a risk factor for TRAS [7,33,39,40]; a retrospective study of 2007-2014 found no relation [3]. Given the diverse information, surveillance is recommended in those patients who present positive serology; in our center, intermediate-risk patients receive prophylaxis with valganciclovir for 90-100 days and those at high risk given for 180-200 days.

The second is factors related to stenosis and its management: the site and degree of stenosis, being more common in anastomosis [3,5], the possible complications during angioplasty that may favor thrombosis, hemorrhage, residual stenosis, and the characteristics of the stent [7,15,35,41,42]. In short stenosis or linear or distal to anastomosis, there are good results only with angioplasty; for longer stenosis or in anastomosis, there are better results when using a stent [7]. In native kidney stenosis, a stent with medication is better to prevent restenosis when compared to a single stent or angioplasties [7,15,22,35,41,42]. In a study, there was a hybrid procedure using both types of stents presenting less restenosis [34]; these patients presented more fibromuscular dysplasia; for this reason, angioplasty with balloon-mounted stents without medication was performed in our patients. We must consider that the flow in and around the stent contributes to the platelet union and to thrombus formation and intimal hyperplasia; there must be a balance between the radial strength and the vessel characteristics to achieve the best late maximum dilation without accelerating the intimal hyperplasia [43]; as a recommendation, 3 mm of the free stent should be placed toward the iliac artery to get a better distribution of the radial strength; remembering that the most common place of stenosis is in the anastomosis, it is also recommended not to use self-expanding stents due to the risk of migration.

Platelet inhibition is recommended given the risk to the acute impairment of renal function by contrast and distal embolisms or the release of atheroembolic particles [44]; these mechanisms of endothelial injury with plate disruption are well studied in the coronary arteries, presenting platelet activation [44,45]. There are some distal occlusion devices to prevent these alterations at the renal level [44]; due to the platelet effects in the genesis of stenosis, stent thrombosis, and the renal injury postangioplasty, we consider to secure a previous anti-aggregation therapy prior to angioplasty at least 10 days according to the half-life of the platelets [46]. It is described that proton pump inhibitors (PPI) and clopidogrel are both metabolized by cytochrome P450 2C19 (CYP2C19) and present polymorphisms and contribute to the decrease of activity [47-49]; in patients with acute coronary syndrome (ACS), the rate of rehospitalization, death, and myocardial infraction may increase [49-51]; some have concluded that there is no difference in mortality and readmissions for ACS or there is no significant difference [52-55]; due to these results, since Hispanic patients are not considered and most focus in Caucasian or Asian patients, as part of the recommendation, it is suggested not to use PPI in patients who are going to undergo an interventional management, opting for other drugs that are not metabolized by this route.

**Figure 1:**
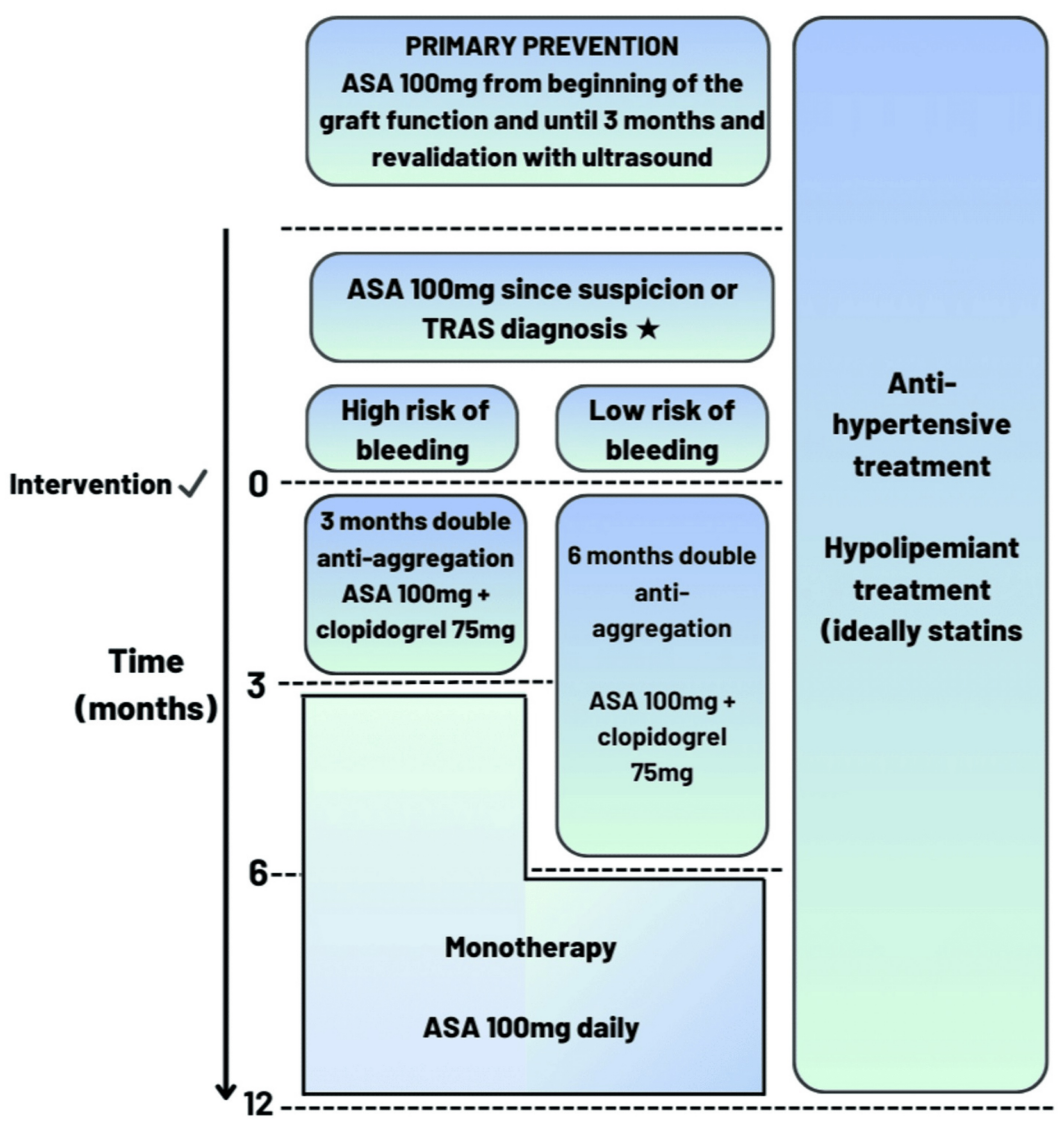
Proposal of treatment ✓Consider suspending PPIs with the usage of clopidogrel ★Consider keeping ASA 100 mg prior to the procedure ASA, acetylsalicylic acid; TRAS, transplant renal artery stenosis; PPIs, proton pump inhibitors

Lastly, the third is the comorbidities presented by these patients, such as micro- and macrovascular complications, calcifying environment, age, other chronic diseases, the duration of the kidney disease type, the duration of renal replacement therapy (RRT), and, finally, the cardiovascular and bleeding risk [3-5]. Once we consider all these factors that may be related to adverse drug reaction (ADR), in addition to an assessment to identify which patients will benefit from conventional therapy for six months or a shortened three-month therapy, an individual assessment of bleeding and cardiovascular risk should also be performed. We therefore propose these two antiplatelet management modalities that can be used as a guide to identify these factors and allow for individualized treatment. We also recommend follow-up and a targeted questionnaire to assess bleeding during treatment. This proposed algorithm is illustrated in Figure 1.

Limitations

The vast majority of the information on the management of the TRAS has been obtained from patients with stenosis in the native kidney, but this is not unified; there are no experimental models or clinical trials that evaluate the efficacy of each regimen of DAPT either in native or transplanted kidneys. Actually, the duration of the different regimens of DAPT in the cardiovascular setting continues with new models and trials that search for the optimal duration, with models that offer shorter duration, but there is an important lack of information in the context of the stenosis of the native and transplanted kidneys. The present work is a proposal of schematic models that help to decide between two regimens of treatment and takes into account the factors that are related to the genesis of the TRAS. As a retrospective study, there are patients who could not be identified; others developed TRAS after one year of follow-up in the institution. Not all the patients had an ultrasound in the follow-up, only when the treating physician considered the circumstance pertinent; the present model could be susceptible to further modifications according to new models of study in the different modalities of antiaggregant therapy.

## Conclusions

Angioplasty with or without stent is a safe procedure for handling patients with TRAS; it is important to mention that transplanted patients, due to underlying disease such as kidney disease, immunosuppression on transplant, and the associated cardiovascular conditions, have a high risk of complications, so the management of an accurate antiplatelet therapy can include primary prevention, some days prior to the procedure and on the same day of the intervention. In addition, the subsequent management is essential in order to ensure good clinical results, such as antihypertensive management and the reduction in elevated serum creatinine and the potential complications prior to angioplasty such as thrombosis and restenosis, among other minor complications such as bleeding in the puncture site and aneurysms.
